# Exploring the effect of implementation and context on a stepped-wedge randomised controlled trial of a vital sign triage device in routine maternity care in low-resource settings

**DOI:** 10.1186/s13012-019-0885-3

**Published:** 2019-04-18

**Authors:** Nicola Vousden, Elodie Lawley, Paul T. Seed, Muchabayiwa Francis Gidiri, Umesh Charantimath, Grace Makonyola, Adrian Brown, Lomi Yadeta, Rebecca Best, Sebastian Chinkoyo, Bellington Vwalika, Annettee Nakimuli, James Ditai, Grace Greene, Lucy C. Chappell, Jane Sandall, Andrew H. Shennan, on behalf of the CRADLE Trial Collaborative Group, Doreen Bukani, Paul Toussaint, Adeline Vixama, Carywyn Hill, Emily Nakirijja, Doreen Birungi, Noela Kalyowa, Dorothy Namakuli, Josaphat Byamugisha, Nathan Mackayi Odeke, Julius Wandabwa, Fatmata Momodou, Margaret Sesay, Patricia Sandi, Jeneba Conteh, Jesse Kamara, Matthew Clarke, Josephine Miti, Martina Chima, Mercy Kopeka, Bellington Vwalika, Christine Jere, Thokozile Musonda, Violet Mambo, Yonas Guchale, Feiruz Surur, Geetanjali M. Mungarwadi, Sphoorthi S. Mastiholi, Chandrappa C. Karadiguddi, Natasha Hezelgrave, Kate E. Duhig, Monice Kachinjika, Mrutyunjaya Bellad, Jane Makwakwa

**Affiliations:** 1 Department of Women and Children’s Health, School of Life Course Sciences, Faculty of Life Sciences and Medicine, King’s College London, London, SE1 7EH UK; 2 Department of Obstetrics and Gynaecology, College of Health Sciences, University of Zimbabwe, Harare, Zimbabwe; 3 Women’s and Children’s Health Research Unit, KLE Academy of Higher Education and Research, Jawaharlal Nehru Medical College, Belgaum, Karnataka 590010 India; 4 Maternity Worldwide, Community Base, 113 Queens Rd, Brighton, BN1 3XG UK; 5 Welbodi Partnership, Ola During Childrens Hospital, Freetown, Sierra Leone; 6 Department of Obstetrics and Gynaecology, Ndola Teaching Hospital, Ndola, Zambia; 7 Department of Obstetrics and Gynaecology, University of Zambia, Lusaka, Zambia; 8 Department of Obstetrics and Gynaecology, Mulago Hospital, Makerere University, Kampala, Uganda; 9 Sanyu Africa Research Institute, Mbale Regional Referral Hospital, Mbale, Uganda; 10 Hope Health Action, Hopital Convention Baptiste d’Haiti, Cap Haitien, Haiti

**Keywords:** Implementation strength, Hybrid trial, Complex intervention, Global health, Maternal mortality

## Abstract

**Background:**

Interventions aimed at reducing maternal mortality are increasingly complex. Understanding how complex interventions are delivered, to whom, and how they work is key in ensuring their rapid scale-up. We delivered a vital signs triage intervention into routine maternity care in eight low- and middle-income countries with the aim of reducing a composite outcome of morbidity and mortality. This was a pragmatic, hybrid effectiveness-implementation stepped-wedge randomised controlled trial. In this study, we present the results of the mixed-methods process evaluation. The aim was to describe implementation and local context and integrate results to determine whether differences in the effect of the intervention across sites could be explained.

**Methods:**

The duration and content of implementation, uptake of the intervention and its impact on clinical management were recorded. These were integrated with interviews (*n* = 36) and focus groups (*n* = 19) at 3 months and 6–9 months after implementation. In order to determine the effect of implementation on effectiveness, measures were ranked and averaged across implementation domains to create a composite implementation strength score and then correlated with the primary outcome.

**Results:**

Overall, 61.1% (*n* = 2747) of health care providers were trained in the intervention (range 16.5% to 89.2%) over a mean of 10.8 days. Uptake and acceptability of the intervention was good. All clusters demonstrated improved availability of vital signs equipment. There was an increase in the proportion of women having their blood pressure measured in pregnancy following the intervention (79.2% vs. 97.6%; OR 1.30 (1.29–1.31)) and no significant change in referral rates (3.7% vs. 4.4% OR 0.89; (0.39–2.05)). Availability of resources and acceptable, effective referral systems influenced health care provider interaction with the intervention. There was no correlation between process measures within or between domains, or between the composite score and the primary outcome.

**Conclusions:**

This process evaluation has successfully described the quantity and quality of implementation. Variation in implementation and context did not explain differences in the effectiveness of the intervention on maternal mortality and morbidity. We suggest future trials should prioritise in-depth evaluation of local context and clinical pathways.

**Trial registration:**

Trial registration: ISRCTN41244132. Registered on 2 Feb 2016

**Electronic supplementary material:**

The online version of this article (10.1186/s13012-019-0885-3) contains supplementary material, which is available to authorized users.

## Background

Despite recent advances, 800 women die every day in pregnancy and childbirth, 99% of which are in low- and middle-income countries (LMIC) [1, 2]. The leading causes of death are haemorrhage, hypertensive disorders and sepsis [2], the majority of which can be prevented with established, cost-effective interventions [3]. Yet, in LMIC, challenges such as inadequate numbers of trained health care providers (HCP) [4] and insufficient access to reliable, accurate, equipment to monitor vital signs [5–8] lead to delays in identifying women with pregnancy complications which contributes to preventable mortality and morbidity [9, 10]. Current priorities of the global health community include combining single effective interventions into packages of care, alongside strategies to improve uptake, coverage and sustainability of these interventions [11, 12].

The success of any intervention is dependent on its use in a specific environment and population [13]. Understanding the most effective routes to deliver these complex interventions and how they may work in varying local contexts is key [14]. Randomised controlled trials (RCT) are often criticised for providing little information about why and how an intervention worked (or not) and the context within which it was delivered [15, 16]. This limits the reproducibility of findings. Sound interventions may be rejected if shown to be ineffective. Knowing which components of an intervention and their delivery are necessary to produce an effect in a certain population is vital for results to be reproduced, adapted or scaled up. This is of even greater importance in low-resource countries with high burden of disease.

Guidance exists on how to evaluate implementation alongside effectiveness [14, 17, 18], integrating mixed-methods to evaluate how well an intervention was delivered, to whom, in which context and how it may work [18]. Hybrid effectiveness-implementation trials aim to evaluate implementation alongside effectiveness [19]. Whilst this methodology is established in the evaluation of health promotion and public health interventions, its application in maternal health in low-resource settings is scarce [20] and few studies are planned [21–25].

The CRADLE-3 trial was a pragmatic, stepped-wedge RCT of a novel vital signs device and training package introduced into routine maternity care, in ten clusters across Ethiopia, India, Haiti, Malawi, Sierra Leone, Uganda, Zambia and Zimbabwe with the of aim reducing a composite outcome maternal death, emergency hysterectomy and eclampsia [26]. The trial was accompanied by a nested mixed-method process evaluation which was informed by the Medical Research Council guidance for complex interventions [18]. The CRADLE Vital Signs Alert (VSA) accurately measures blood pressure (BP) and heart rate, calculates shock index (heart rate divided by systolic BP) [27–30] and displays results on a traffic light early warning system which indicates abnormal vital signs (Additional file 1: Figure S1) [31]. This is important in LMIC where routine clinical tasks, such as vital signs measurement, are often undertaken by those with minimal training, and community health workers also play a vital role in maternity care, often being the first point of contact and an essential link to clinical services [11, 32].

**Fig. 1:**
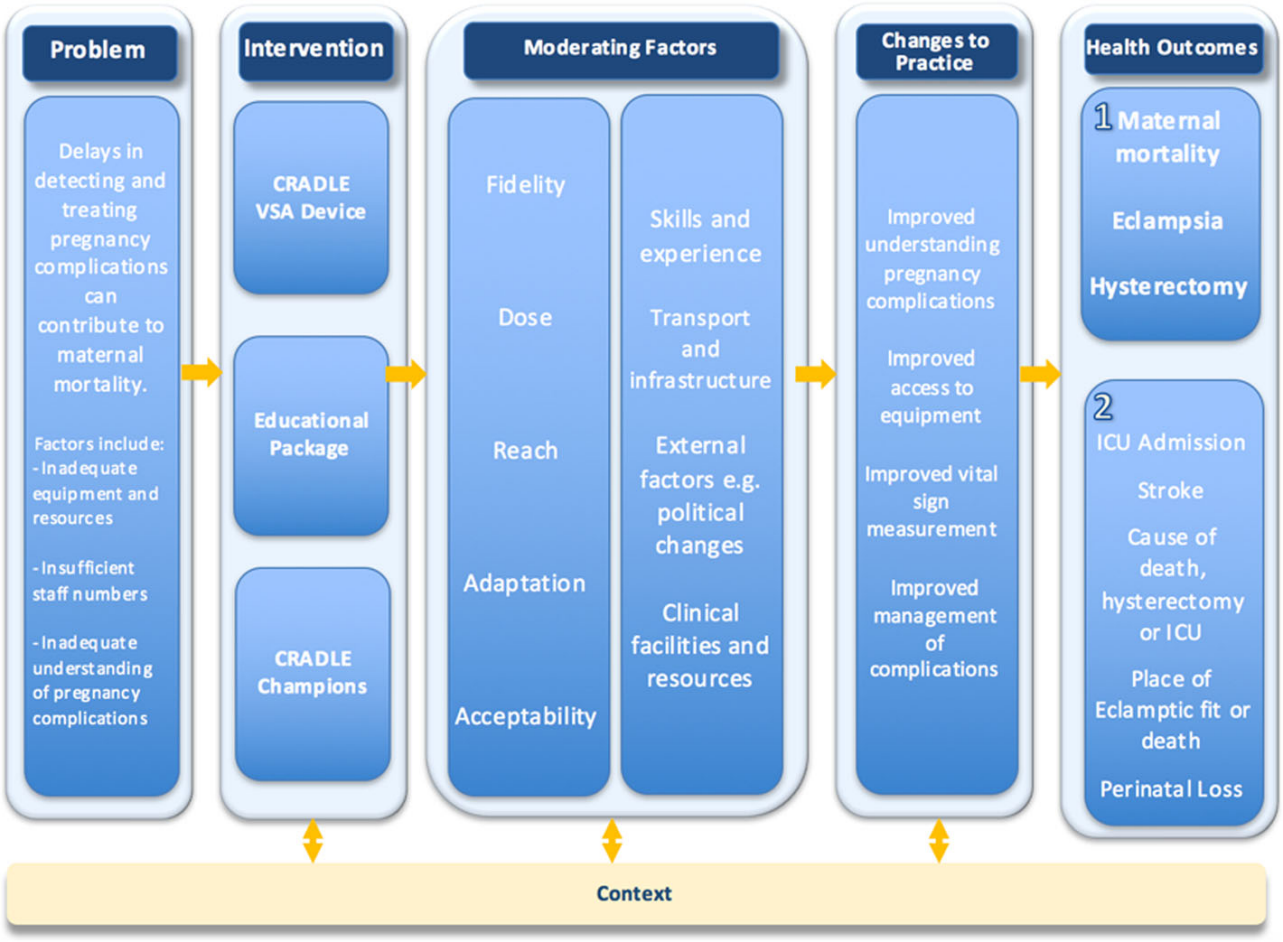
Logic model for the CRADLE intervention

It was hypothesised that better availability of equipment would improve the efficiency and capacity of HCP to monitor vital signs. It was also hypothesised that training would improve HCP understanding of when and how to measure vital signs and how to identify and manage pregnancy complications. The ease of use of the CRADLE VSA and the traffic light early warning system would mean that all cadres of HCP would be alerted to abnormal vital signs. Together, this would result in more women receiving more vital signs measurements, so abnormal results would be identified earlier and managed faster, thus reducing maternal morbidity and mortality. These hypotheses were developed through field studies, stakeholder engagement and literature demonstrating need for improved access to equipment [5–8] training in detection and management of pregnancy complications [33–35] and task-sharing [36, 37] in maternity care in low-resource settings. In addition, qualitative evaluation [31] and a mixed-methods feasibility study [38] determined that the device is robust and easy to use by any cadre of HCP and that the training package and implementation strategy were acceptable and had potential to impact on clinical management (escalation and referral). A logic model was created (Fig. 1) to present these assumptions, processes and anticipated outcomes. This informed the key areas for evaluation in this study.

Our aim was to describe the implementation of the intervention and the local contexts in which it was delivered and to determine whether differences in the effect of the intervention on the primary outcome can be explained. This can be divided into several objectives informed by the RE-AIM framework [15, 39]. These were chosen with the aim of exploring if and how this pragmatic intervention impacted on routine maternity care in a wide variety of settings:

- To evaluate whether the intervention was implemented as outlined in the protocol by describing the quantity and quality of training in each setting.
- To determine the reach of the intervention by evaluating the extent to which health care professionals and women were exposed to the intervention.
- To explore how the intervention was adopted into routine maternity care, whether this changed over time and the potential sustainability of this.
- To explore differences in context, implementation, reach and adoption between sites and determine whether they can explain differences in the effect of the primary outcome in different settings.
- To explore if and how the intervention impacted on routine maternity care across the facilities in each setting and identify possible reasons for this.

## Methods

### Intervention

The intervention comprised the CRADLE VSA delivered through a one-off interactive training session of CRADLE Champions. These were purposely selected HCP from each ward or facility in the trial cluster. They were selected prior to implementation, either as managers and/or as influential in their clinical area by the local research team. Interactive training sessions covered the use and maintenance of the device and suggested clinical management in response to abnormal vital signs using presentations, demonstration, practice and clinical scenarios. The CRADLE Champions were provided with posters, training manuals and a short, animated training film (sent by Bluetooth to smartphones). The CRADLE Champions then used these materials to provide ongoing training and support in their clinical area. These components of the intervention and implementation were developed during a 6-month feasibility phase with input from stakeholders [38]. The local research team continued to provide regular support to all facilities with at least monthly contact. Existing equipment for measuring vital signs was usually removed from clinical use, unless it had a specific function such as automation for high dependency. This intervention was compared to routine maternity care using locally available medical devices and management guidelines [26, 40].

### Design and setting

Each cluster comprised at least one urban or peri-urban secondary or tertiary health facility that provided comprehensive emergency obstetric care with multiple peripheral facilities that refer to the central hospital [26]. The stepped-wedge design meant that clusters crossed over from control to the CRADLE intervention in one of nine steps at two monthly intervals over the 20-month trial duration. The order of steps was randomly allocated using a computer-generated sequence [26]. This design was chosen to minimise the risk of bias and show causality, should a significant effect of the intervention be demonstrated.

### Population

All HCP working in maternity care in the cluster facilities had access to the intervention including community HCP in two clusters where they were active in routine maternity care and approved for inclusion (Ndola and Cap Haitien). All women identified as pregnant or within 42 days of delivery, that presented to routine maternity care, were exposed to the intervention without exclusion.

### Outcomes

**Fig. 2:**
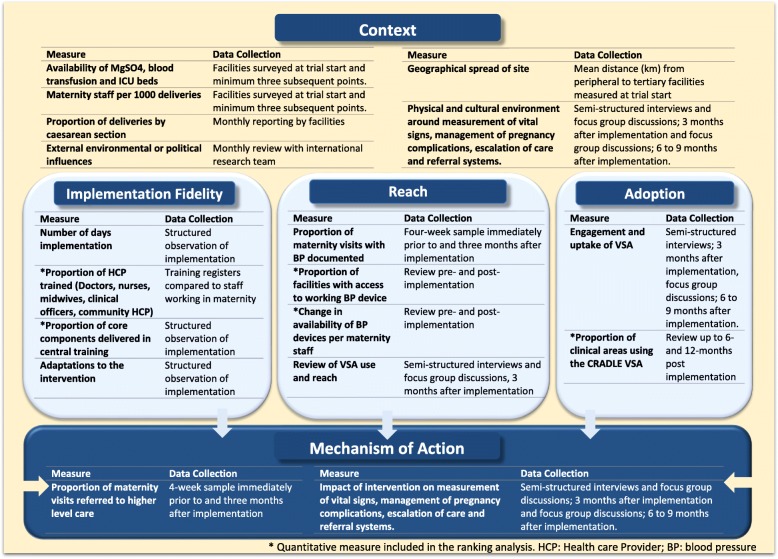
Implementation domains and methods of data collection. Asterisk denotes quantitative measure included in the ranking analysis. HCP health care provider, BP blood pressure, VSA Vital Signs Alert

The primary outcome was a composite of at least one of maternal death, eclampsia or emergency hysterectomy per 10,000 deliveries. The implementation and impact of the intervention in each site was evaluated by mixed-methods under three implementation domains as shown in Fig. 2, informed by the RE-AIM framework [15, 18, 41]. We identified potential ways in which the intervention may be working, and the necessary resources and actions required for this, then selected measures that were important but feasible to collect within this pragmatic, multi-centre trial design [38].

### Data collection

Baseline data were collected from each facility on the distance from the nearest tertiary referral hospital; number of HCP working in maternity (doctors, nurses, midwives, clinical officers and community HCP in Ndola and Cap Haitien); availability of existing BP equipment; blood transfusion services; intensive care beds; and magnesium sulfate. These were selected as markers of health system context that were important and feasible to measure. This was updated a minimum of three times during the trial period. Major changes to the political or physical environment such as infrastructure, staff retention and extreme weather conditions were evaluated monthly. The number of deliveries in each cluster was collected by review of facility registers and routine reporting. Community deliveries were captured through a variety of methods such as household visits from community health workers in India and monthly reporting meetings with traditional birth attendants in Haiti (three sites did not routinely record deliveries that occur outside of facilities).

Training was observed against a pre-defined observational checklist, including the number of training days and the proportion of core content delivered. Training registers were completed and compared to staffing numbers. All clusters reported at six monthly intervals on the proportion of clinical areas using the CRADLE VSA device. In order to evaluate the ways in which the intervention, and participants interaction with it, may trigger change (mechanisms of action) [38], the number of women attending maternity services, the proportion that had their BP measured and the proportion referred to higher level care were measured for a 4-week period immediately prior to implementation and 3 months after implementation. This was integrated with qualitative findings on context and use of the device.

In each site, we undertook semi-structured interviews (*n* = 3–5) and focus group discussions (*n* = 1) with HCP, 3 months after implementation. These explored the uptake of the intervention, its influence on clinical management and any unexpected consequences. In sites that implemented in the first 14 months of the trial, a further focus group discussion was undertaken at 6–9 months after implementation to explore whether influence on clinical management, escalation and referral systems changed over time and the sustainability of the intervention. In total, we conducted 36 interviews and 19 focus group discussions with 130 participants across the ten sites. Participants were selected through purposive sampling to ensure representation of different HCP cadres and facilities. Participants were approached face-to-face and gave written informed consent. These were recorded, and transcribed verbatim and field notes were recorded. Content and notes were reviewed iteratively to identify further participants until data saturation was achieved. All qualitative work was undertaken, translated and transcribed by experienced local research coordinators (with clinical background) following training from the trial coordinator and senior social scientist (JS) or qualitative researchers. Researchers had limited prior relationship with the participants. Two data coders that were independent to the interviewers undertook initial analysis using QSR NVivo 11 software (QRS, Vic, Australia) prior to revealing the analysis of the primary outcome then further analysis once the results were known. We used the framework method with a coding framework that drew upon the study objectives, logic model and interview guide [42, 43]. New concepts initiated by participants that could not be categorised were coded using an inductive approach [44].

In order to compare implementation and determine whether this was related to effectiveness, we used a ranking approach as previously described in other fields [45–47]. Clusters were ranked from highest to lowest on selected quantitative outcomes on implementation fidelity, reach and adoption (marked by an asterisk Fig. 2). These were selected as the direction of benefit was clear, whereas the anticipated direction of change for outcomes on context and action were less clear (e.g. poorer availability of resources at the trial start may be associated with greater benefit from the intervention due to greater need, or less benefit due to inability to respond to abnormal vital signs). Outcomes under the same domain were averaged and converted to a possible range (0 to 1) to give each cluster a score for each domain analysed (implementation fidelity, reach and adoption). These were then averaged to give each cluster a single composite score reflecting their implementation (possible range 0–1) [45–47]. Due to the stepped-wedge design, the single measure of adoption was only available in eight of the ten sites. The individual domain scores and overall composite score were compared to primary outcome in each site. Correlation between the individual measures within domains was also determined [48].

### Statistical analysis

Statistical analyses were undertaken in Stata version 14.2. For the primary outcome in individual sites, the main analysis used logistic regression with generalised estimating equations and a population-averaged model. Adjustments were made for fixed centre effects (categorical) and separate fixed linear trends (continuous) in each centre to account for changes in the primary outcome over time [49]. Results are reported as odds ratios (ORs). Details of randomisation and further analysis of the trial are published in protocol [26] and primary results paper [50]. For the evaluation of implementation, the ranks were summarised, and simple rank correlations calculated. We used meta-regression to see if the primary outcome in individual sites were related to the individual and composite implementation scores [51]. For comparison of referral rates before and after implementation, unadjusted OR were calculated and combined using random effects meta-analysis [52]. In each site a 4-week period immediately prior to and 3 months after implementation were compared; this is a non-randomised comparison.

## Results

### Implementation fidelity

The average duration of implementation training across all facilities was 10.8 days (range 7 days in Addis Ababa to 18 days in Mbale). In total, 2747 HCP were trained, 61.1% of all those working in maternity services in those sites (range 16.5 in Kampala, Uganda to 89.2% in Zomba, Malawi, Table 1). Nine of the ten sites delivered all the key content of training. Freetown, Sierra Leone was the first to implement with less emphasis on training senior staff, the background of device development and validation studies. Following challenges from senior staff in accepting device accuracy, this was emphasised in subsequent site training.

**Table 1:** Quantitative implementation measures of implementation fidelity and reach

	Implementation	Reach					Adoption			
Site	Staff trained in roll-out period	Proportion of women with BP measurement pre-intervention^ψ^	Proportion of women with BP measurement post-intervention^ψ^	Unadjusted comparison	Facilities with working BP pre-intervention	Facilities with working BP post-intervention	Clinical areas using solely VSA at 6 months	Clinical areas using VSA with other devices at 6 months	Clinical areas using solely VSA at 12 months	Clinical areas using VSA with other devices at 12 months
	*N* (%)	*N* (%)	*N* (%)	OR (95% CI)	% (devices, HCP)	% (devices, HCP)	%	%	%	%
Addis Ababa	192 (36.9%)	*	*	*	100% (1:3)	100% (1:2)	33.3%	66.7%	υ	υ
Cap Haitien	189 (73.5%)	*	*	*	100% (1:3)	100% (1:3)	76.2%	23.8%	υ	υ
Freetown	243 (57.9%)	2199 (87.7%)	3335 (100%)	1.14 (1.12–1.16)	84·6% (1:13)	100% (1:4)	73.1%	26.9%	96.2%	0.0%
Gokak	297 (87.1%)	*	*	*	97·5% (1:1)	100% (1:0.7)	72.3%	27.7%	υ	υ
Harare	405 (69.9%)	*	*	*	92% (1:12)	100% (1:5)	υ	υ	υ	υ
Kampala	188 (16.5%)	*	*	*	92.3% (1:19)	100% (1:4)	33.8%	47.9%	34.2%	47.9%
Lusaka	265 (33.9%)	*	*	*	100% (1:10)	100% (1:5)	75.9%	24.1%	υ	υ
Mbale	314 (59.2%)	4640 (42.6%)	11,300 (96.2%)	2.26 (2.21–2.31)	92% (1:5)	100% (1:2)	87.3%	9.9%	υ	υ
Ndola	349 (86.6%)	5673 (98.2%)	5782 (100%)	1.02 (1.0–1.2)	93.5% (1:7)	100% (1:2)	90.2%	9.8%	90.2%	9.8%
Zomba	305 (89.2%)	11,860 (88.4%)	10,783 (94.1%)	1.06 (1.06–1.07)	100% (1:17)	100% (1:2)	υ	υ	υ	υ
Total/Mean	2747 (61.1%)	6093 (79.2%)	7800 (97.6%)	1.30 (1.29–1.31)	95% (1:9)	100% (1:3)	73.1%	23.3%	73.5%	20.0%

*Data not recorded; *BP* blood pressure, *VSA* Vital Signs Alert

^υ^Data not available due to stepped wedge trial design (time point exceeded trial duration)

^ψ^Recorded for a 1-month period immediately prior to implementation, and a 1-month period 3 months after implementation

Educational materials were translated (India, Ethiopia, Malawi, Haiti), and delivery was adapted to take into account locally available medications and referral structures. In India, all training was delivered by the research team rather than via CRADLE champions (87.1% trained). In Haiti, community HCP without formal training had a longer duration of training (approximately 2 days), spending more time checking understanding. The duration of training was longer in sites with a wider geographical spread or more challenging terrain (Mbale, Uganda; 18 days and Zomba, Malawi; 16 days) except in India, which was able to mobilise a larger local research team (10 days). External events influenced implementation in two sites. One of three tertiary hospitals in Cap Haitien, Haiti was closed at the time of implementation due to strike action, therefore key managers were trained, and remaining staff received training within 2 weeks of opening. In Ndola, Zambia, implementation coincided with roll-out of alternative (un-related) training for some maternity staff by the Ministry of Health. Implementation went ahead as planned for remaining staff, and those that were unable to attend were trained by champions or the research team in the subsequent week.

Clusters that trained fewer staff tended to have multiple, very large facilities with high numbers of deliveries (Lusaka and Kampala), except Freetown, which was a smaller unit but trained fewer staff. This cluster was the first to implement, possibly demonstrating the learning curve of the research team. Qualitative findings demonstrated that the majority of participants from all sites felt the training was adequate (demographic details of the qualitative participants are shown in Table 2). Champions felt confident using the materials to orientate their colleagues. Recipients of training from champions were confident to use the VSA and also to orientate others. A small minority of participants from the three sites that trained the fewest HCP (Addis Ababa, Kampala and Freetown) highlighted that training from the champions had been brief, that staff who were not trained took longer to learn and faced initial challenges with use, or that ongoing training may not be sustainable with staff turnover (quotes to illustrate in Table 3).

**Table 2:** Demographic details of interview and focus group participants

	Interview participants	Focus group discussion participants	Focus group discussion participants
Time after implementation	3 months	3 months	6 to 9 months
Mean duration (minutes)(range)	25 (7–45)	64 (39–125)	58 (35–87)
Profession			
Midwife	16	38	28
Nurses	13	23	18
Doctors	3	2	4
Clinical officers	0	1	0
Community HCP	1	4	5
Allied HCP	3	2	5
Gender			
Male	5	7*	7
Female	31	57*	53
Age (years)			
18–24	2	0*	0
25–34	12	24*	20
35–44	14	31*	23
45–54	6	7*	8
55–65	2	2*	9
Educational level			
Secondary or less	2	8*	6
Diploma	27	47*	30
Certificate	2	5*	11
Bachelor’s degree or higher	5	4*	13
Experience (years)			
1–5	12	16*	15
6–10	8	17*	13
11–15	9	16*	14
> 15	7	15*	18

*Demographic detail missing for 6 participants from one focus group discussion in Malawi

**Table 3:** Selected quotes to illustrate qualitative themes

Domain	Theme	Qualitative quote
Fidelity	Quality of training	Midwife, Tertiary Hospital, Ndola, Zambia: “Sister* who had an opportunity to attend the one-day workshop where the orientation was done (on) how to use the BP machine and she is the one who disseminated the information, orientated myself and other midwives from labour ward on how to use this BP machine … after I got used I have not had any problems and I have also oriented other medical personnel on how to use it. It has been so helpful.”
		Midwife, Clinic, Addis Ababa, Ethiopia: “The apparatus is very good. But there was a minor problem. None of us took any training, but instead we just received the apparatus and we started putting it to use. And it had some problems on accuracy.”
Reach	Better availability of equipment meant more measurements done and on more women.	Clinical Officer, Peripheral Hospital, Zomba, Malawi: “it’s just a matter of having enough resources now, that at least we have a BP machine in the ward and outside to the health centers, and indeed is the easy one, is the fast one. So this is what can take at the same time to listen to the BP. So the things I see at least is that the BP are taking place more often, because the BP machine is always available”
	Better availability of equipment reduced time taken to monitor.	Midwife, Outpatients, Tertiary Hospital, Freetown Sierra Leone: “… with one referral the pressure was down, it prompted us greatly, to start IV fluids, call a doctor to come back from theatre...(details of clinical management). This machine (VSA) helped us a lot because if it was any other machine where we use stethoscope, we would have needed to run around trying to look for it, but that machine is already there. That morning the machine saved her, because thank god she survived, was discharged, and we are happy about that.”
	Increased confidence resulted in increased monitoring in facilities	Medical Officer, Primary Health Centre, Gokak, India: “Earlier, because of overload of work our ANMs were not checking the BP with mercury apparatus. Now, after introduction of this machine and after showing this to AHSAs also in a meeting, they are happy with what they are doing. And they are doing more checks than they were doing earlier. Earlier, they were sending pregnant women to the doctor, telling them that the doctor will check the BP. But now they are also checking and then sending the patients to me.”
	Task-sharing increased monitoring in communities	Registered Nurse, Clinic, Ndola, Zambia: “ … we had a case last month where a SMAG member (Safe Motherhood Action Group volunteer) got BP machine to go and visit a woman in one of the villages and she discovered that BP was high and client referred and managed at Ndola Central Hospital. Without CRADLE that woman would have been left to die in the community. The CRADLE VSA machine is helping us a lot.”
Adoption	Ease of use	Junior midwife, Clinic, Addis Ababa, Ethiopia: “In our health center, the former device used to fail often. This one measures both the blood pressure and pulse. This means it helps us forecast to the future, about the condition of the woman. Before this, we used to measure only blood pressure, and that’s it. But now since it helps us forecast her pulse. It assesses the condition of the woman, whether she is entering a danger zone or not. It helps us to take care of the mother before the event. Second, since it helps us with the management during the emergency case, it prepares us to take action immediately. So, it guides us, it measures pulse and as long as we follow the rules of positioning, its accuracy is also very good. I like it.”
	Improved reliability and champion/research team support	Midwife, Clinic, Addis Ababa, Ethiopia: “The devices we used before were very prone to malfunctioning. But if this one malfunctions, it is often due to improper usage. When such problems happen call (the research assistant) and try to solve the problems. In general, it is helpful for our health center.”
	Security	Midwife, Labour Ward, Tertiary Hospital, Ndola, Zambia: “Yes we had one experience. I think they were just introduced. One blood pressure machine had gone missing … And from that time we learnt a lesson. So each time we are changing shift all the equipment is put on the table and handed over.”
Mechanism of action	Cultural change	Nursing officer, Clinic, Mbale, Uganda: “it has become a quality issue to monthly charts on how many patients had their BP taken each month since machines are readily available for use. It has increased the work load since every mother has to have her BP taken.”
	Alerts trigger increased investigation	Maternity Health Assistant, Clinic, Freetown, Sierra Leone “… we check them all when they come and if I see those colours I call my boss. Because when the machine shows those colours it’s a problem and we need to ask her some questions. And if she says she’s not well I can say there is something wrong with her. It will not show this colour if they are well. A person who is well will show a green colour. And if it still persists for two or three times with that colour, we can send them to do tests sometimes to detect anaemia, sometimes (haemoglobin) 9 grams or 8 grams. So we can treat her little by little.”
	Alerts aid communication	Midwife, Private Labour Ward, Ndola, Zambia: “as a midwife, as nurse you are supposed to know the abnormal so because of that at least it has … I would not say it has changed (management) much but sometimes yes it does help us. When it is red we do the BP we check again, if its red it means the BP is very high we have to inform the doctor immediately. So there are times when you would call the doctor and they not in, you make sure you emphasize because that the patient has to be seen soon..”
	Alerts convince women and families	Midwife, Clinic, Ndola, Zambia: “if it shows red, I also explain to her even showing her to say “have you seen this colour? Where is this arrow pointing, no it is pointing up. Yes, this means am supposed to refer you to Ndola central” but like before when we were using the digital and the mercury we would just do and even if the patient reads the readings to be high (they) would not understand. But this time around since it has colours, even for somebody who is not learned, it is easy to convince them”
	Alerts motivate women	Auxillary Nurse Midwife, Community Health Post, Gokak India: “The number of those who come themselves voluntarily has increased and the awareness of getting the BP checked has improved among them. If we show them the reading, they think “Yes, all such things are there in the BP; we need to get checked; if it shows yellow it means this; if it shows red it mean this, there are the signals for us”.”

### Reach

Overall, 3868 devices were delivered across 286 facilities. Four clusters recorded the proportion of women with BP measurement. All demonstrated a significant increase in measurements made after the intervention (usual care mean 79.2% (*n* = 6093/7693) vs. intervention 97.6% (*n* = 7800/7992); OR 1.30, 95% CI 1.29–1.31); Table 1). Prior to the intervention, 95% of facilities had access to at least one working BP machine. After the intervention, 100% had access, with better availability per HCP in all clusters. Participants from both clinics and hospitals in every cluster except Haiti reported an increase in the availability of equipment. The availability of equipment, and its ease of use, meant that more vital signs measurements could be done and faster, as staff did not spend time looking or waiting for equipment (Table 3).

Many participants reported that students and other allied HCP or volunteers would regularly help to take vital signs measurements with the device. More junior staff also took more vital signs measurements, where they would previously have referred the patient to other HCP for routine monitoring. This was reported to be due to greater confidence in their capacity to measure BP and interpret results. It was frequently commented that this made it more likely that women would have their vital signs measured (Table 3). In Haiti and Ndola, community HCP reported confidence and pride in being equipped and skilled to monitor vital signs in their community. This also led to more vital signs measurement in the community and earlier detection of abnormalities (Table 3). A minority of HCP reported that demand still outweighed supply, even though this was improved.

### Adoption

The majority of sites reported rapid use of the device on all pregnant women. The reasons for rapid adoption differed according to site context. Sites with poor availability or poor-quality existing equipment (e.g. Kampala, Freetown, Mbale and Zomba) reported rapid use, irrespective of the different proportion of staff that were trained. Sites with adequate availability of equipment prior to implementation (Gokak and Addis Ababa) elected to use the VSA in preference to other equipment citing ease of use, better accuracy and easier interpretation due to the traffic light alert, which reduced the workload. This was true across all cadres of HCP from community volunteers to medical officers in hospitals.

Due to the stepped-wedge design, eight clusters reviewed use at 6 months post-implementation and three at 12 months. The majority of clinical areas were using solely the CRADLE VSA device at 6 months (73.1%; range 33.3% in Addis Ababa, Ethiopia to 90.2% in Ndola, Zambia, Table 1). Only 4.8% of clinical areas had chosen to use previously existing vital signs devices in preference to the CRADLE device. This was still reflected at 12 months (73.5% using solely the CRADLE device). A minority of sites reported barriers to adoption, the most frequent was the sensitivity of the VSA to movement and positioning, in some cases leading to mistrust of the accuracy of results. This was reported more frequently in sites with low fidelity (Freetown, Kampala and Addis Ababa). However, qualitative findings in Freetown suggest that active support from the champions or the research team resolved this concern, and this correlated with improved adoption compared to Addis Ababa and Kampala and over time (Table 1).

By the trial end, 4·6% (*n* = 180) of VSA were reported to be broken. The most commonly reported reasons were failure of the battery, leaking of the valve in the pump or tears in the cuff. Many sites noted it was more robust than pre-existing equipment (Table 3). Very few CRADLE VSA were reported missing by the trial end (0·6% (*n* = 23). Sites described self-directed systems of handover or registration to minimise this risk (Table 3).

### Relationship with clinical outcome

**Fig. 3:**
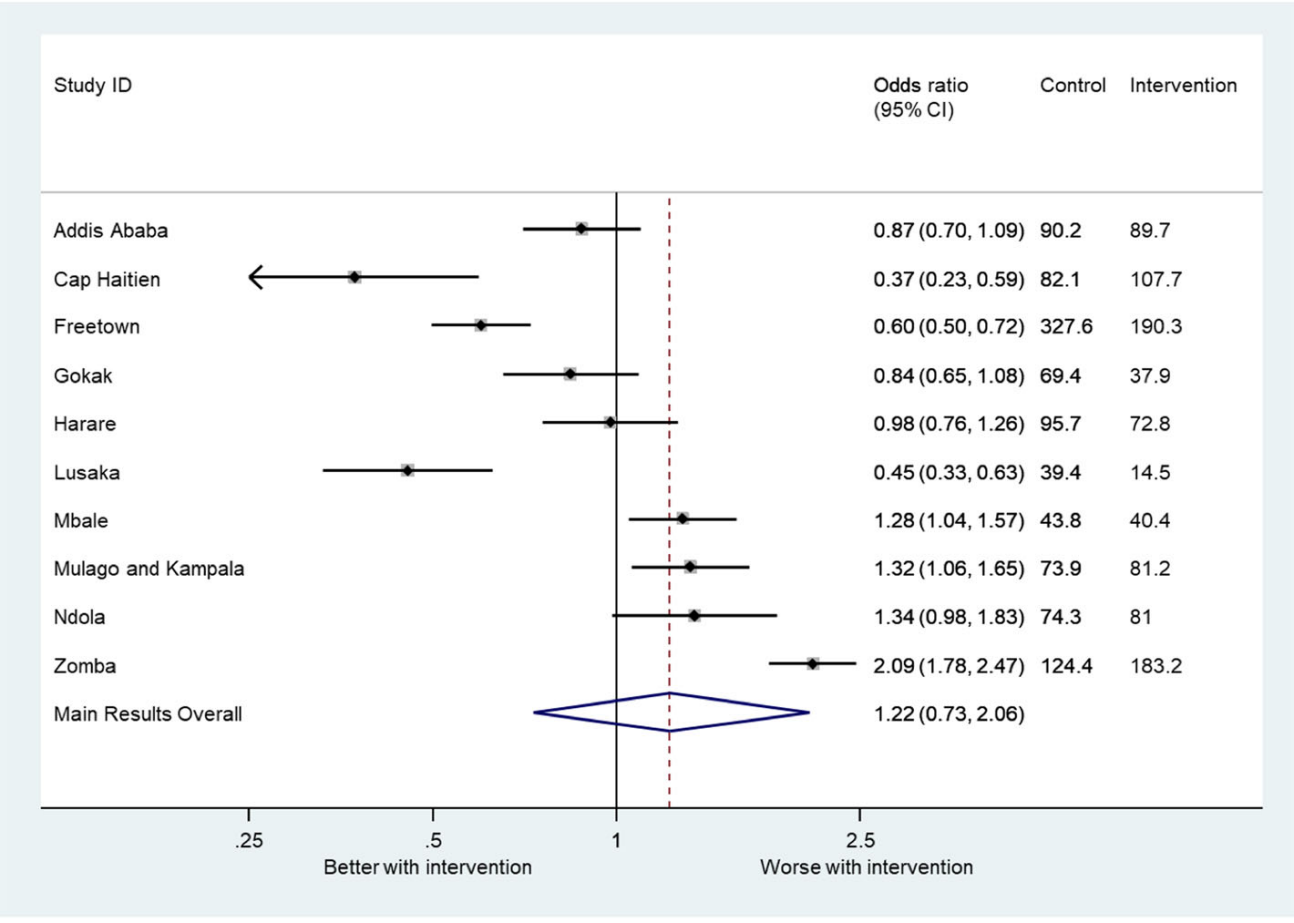
Forest plot showing odds ratio for primary outcome in individual clusters in the intervention period compared to the control period

The effect of the intervention on the primary outcome is shown in Fig. 3 (Additional file 1: Table S2). After planned adjustment for temporal trends, significant benefit of the intervention was shown in Freetown, Cap Haitien and Lusaka, which included the sites with the lowest and highest baseline primary outcome event rate (39.4/10,000 deliveries in Lusaka; 324/10,000 deliveries in Freetown). There was also considerable variation in the implementation, reach, adoption and context between clusters with no significant correlation between the individual measures within any domain, including physical context. There was no significant correlation between the randomised order of implementation and the primary outcome.

The two clusters that trained the highest proportion of staff with the highest content as planned in the protocol (fidelity) were Gokak and Zomba. There was no correlation between fidelity and effectiveness (OR 0.55; 0.19–1.55). The two sites that had the best improvement in availability of equipment (reach) were Freetown and Kampala. Overall, no correlation was demonstrated between reach and effectiveness (OR 0.62; 0.27–1.42). The majority of facilities were using the CRADLE VSA device either alone or in combination with another device at 6 months, and this measure (adoption) was not correlated with the primary outcome (OR 1.40; 0.64–3.04). When domains were aggregated into a composite score, the combination of fidelity, reach and adoption was not significantly associated with the primary outcome (OR 0.93; 0.07–13.01).

### Context and mechanism of action

Across all clusters an average of 50.0% of deliveries occurred in the central referral facilities (mean = 1358 per month per cluster), 45.7% in peripheral facilities (mean = 1241 per month per cluster) and 4.3% at home (mean = 118 per month per cluster from seven clusters where this was systematically collected; Table 4). The mean proportion of deliveries by caesarean section was 17% (*n* = 91,158/536,223; range 9–31%). The availability of key obstetric resources and staffing levels are shown in Table 4. In the majority of sites, one staff member (or less) per 1000 deliveries per month joined or left the workforce in each cluster. Availability of magnesium sulfate and blood transfusion services changed in less than 2% of facilities per month in all clusters. The measures of physical context were variable between and within sites. Lusaka, Zomba and Kampala had the fewest total staff per 1000 deliveries. The lowest proportion of caesarean deliveries were done in Lusaka (9%) and Ndola (10%). Ndola also had the lowest proportion of facilities with blood transfusion capacity (6.5%), and Cap Haitien had the fewest facilities with magnesium sulfate (25%). There were a number of external influences during the trial period, for example strike action in Kampala, Uganda and an earthquake outside of the research area in Haiti. However, sites reported minimal impact of these events on care provisions.

**Table 4:** Description of clusters

Site	Central referral facility deliveries	Peripheral facility deliveries	Home deliveries^α^	Proportion of caesarean sections	Capacity for blood transfusion^Ω^	Adult intensive care unit beds^Φ^	MgSO4 availability^Ω^	Total doctors in maternity^Ω^	Clinical officers working in maternity^Ω^	Other HCP working in maternity^Ω^	Distance from peripheral facilities to nearest tertiary facility
	Average per month: mean (SE)	Average per month: mean (SE)	Average per month: mean (SE)	Average % (SD)	Mean % of facilities	*n* per 1000 deliveries	Mean % of facilities	Mean/1000 deliveries	Mean/1000 deliveries	Mean/1000 deliveries	Mean (km) (SD)
Addis Ababa	1114 (144)	657 (132)	–	22% (3)	15.8%	7.4	87.4%	31.2	104.1	154.1	4.3 (2.7)
Cap Haitien	682 (211)	0 (0)	63 (32)	20% (3)	25%	0	25.0%	99.4	13.2	73.1	14.2 (5.4)
Freetown	704 (148)	403 (114)	84 (28)	14% (3)	7.7%	0	100.0%	28.9	15.8	329.6	7.5 (3.7)
Gokak	952 (129)	188 (25)	4 (2)	31% (2)	25%	24.5	41.4%	84.4	1.2	209.9	74 (16.9)
Harare	1026 (64)	785 (53)	108 (21)	22% (25)	12.0%	4.7	100.0%	54.8	1	246.5	16.3 (9.6)
Kampala	2223 (290)	4168 (393)	–	22% (3)	38.5%	2.7	61.5%	20	2.2	129.6	8.7 (4.3)
Lusaka	2231 (457)	3507 (582)	436 (60)	9% (1)	14.1%	3.3	79.8%	21.4	25	78.6	3.3 (1.3)
Mbale	1442 (152)	1583 (90)	–	12% (2)	31.7%	0	78.6%	24.1	39.4	116.6	19.6 (12.4)
Ndola	500 (38)	797 (55)	46 (10)	10% (1)	6.5%	4.5	77.4%	23.1	17.7	126.9	11.3 (5.1)
Zomba	2705 (394)	318 (147)	86 (21)	14% (2)	75%	5·3	95.4%	5	36.9	95.9	68.3 (35.2)
All sites	1358 (766)	1241 (1392)	118^1^ (137)	17% (11)	25.1%	10.9	74.7%	39.2	25.6	154.1	22.75 (19.36)

^α^For the seven clusters that were able to systematically record home deliveries

^Φ^Intensive care unit is defined as a separate ward or room offering higher level care than the main ward

^Ω^Recorded at trial start and a minimum of three times during trial period and average presented

In addition to the mechanisms previously described (better availability of equipment, ease of use and confidence of all cadres of HCP to measure vital signs), the increase in equipment and training meant it was no longer acceptable to not measure vital signs on every woman. Staff reported increased motivation and interest in vital signs measurements. Only one site (Mbale) reported this in a negative light, since measurement of BP on all women increased workload. The other sites reported a reduced workload as time taken to find equipment, measure vital signs and interpret results was reduced, and this task could be undertaken by a wider number of HCP.

It was frequently reported that the intervention prompted HCP to do more investigations, more quickly. This was reported to be because the traffic light display alerted users to results outside the normal range, and HCP had more confidence in the results so were better able to make decisions. This finding was not dependent on the number or skill level of staff. A minority of participants opposed this view, stating that the management was unchanged, as vital signs were always measured and acted upon. This was most commonly reported by senior HCP working in better-resourced environments. Even in this setting, benefit was still reported from the traffic light alert in aiding communication between HCP.

The majority of sites also reported that the alerts were easily understood by women and untrained staff such as ambulance drivers. This was beneficial in conveying the need for management or referral, especially in sites where this was reported to be a key barrier to care (Gokak, Ndola, Zomba, Harare). Some sites reported that increased awareness of vital signs in the community resulted in increasing demand for measurements to be done (Table 3).

**Fig. 4:**
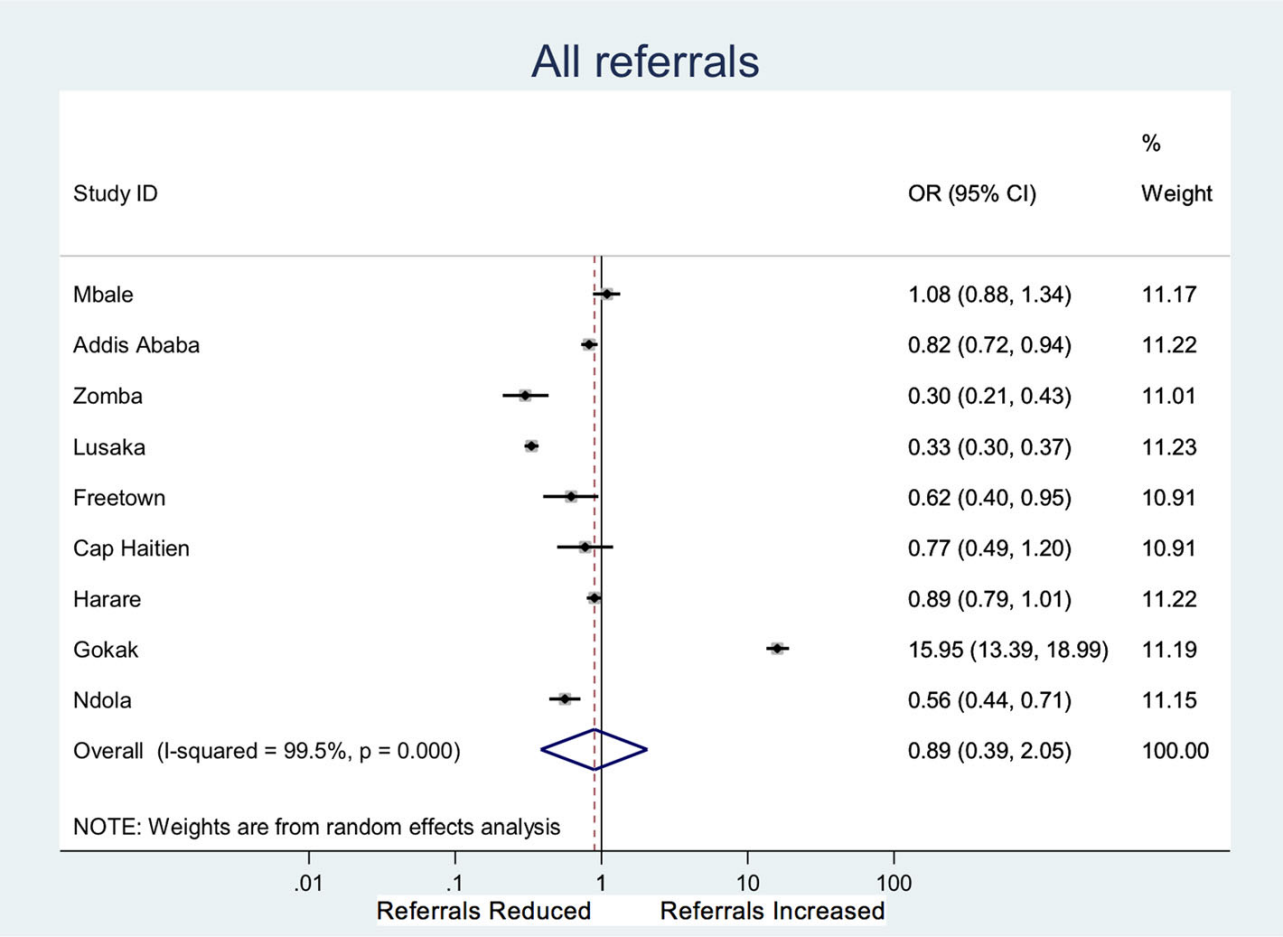
Forest plot showing odds ratio for referral in individual clusters (Data for nine sites that were able to collect denominator data. Data collected for a 4-week period immediately prior to and 3 months after implementation) in the intervention period compared to the control period

The impact on referrals differed between sites. Overall, 3.7% (*n* = 2784/74,828) of women seen in peripheral maternity facilities were referred to higher level care in the control period compared to 4.4% (*n* = 3212/73,371) in the intervention period (OR 0.89; 0.39–2.05) (data for nine sites that were able to collect denominator). However, the majority of sites demonstrated a small but significant reduction in referrals with a single site (Gokak) demonstrating a 16-fold increase (Fig. 4, Additional file 1: Table S1). Qualitative findings suggest the increase in Gokak was a result of increased community monitoring, increased confidence in peripheral HCP to detect abnormal vital signs and convince women to attend, alongside rigorous adherence to referral protocols from rural health posts (subcentre) to primary care centres, meaning all women with asymptomatic anaemia triggering a yellow light were referred. This is in combination with an effective ambulance system, and further systems in place to cope when ambulance services were delayed, to transfer patients from primary care clinic to hospital when acute complications were detected. Therefore, the wide geographical distance (mean 74 km from peripheral clinic to tertiary hospital) of this site did not impede delivery of care.

In contrast, Haiti reported no change in the number of referrals but that abnormal vital signs were detected and referred faster by using the traffic light alerts to convince women to attend, where cultural acceptability and perceived quality of hospital care was a barrier. However, despite the relatively small cluster size (mean 14 km from peripheral clinic to tertiary hospital), the qualitative data indicated that the lack of ambulance service or funds (personal or within the health care facility) to pay for transport led to long delays contributing to morbidity and mortality, irrespective of the capacity to monitor and escalate care peripherally.

Differing acceptability of referrals and the relationship between peripheral and tertiary facilities arose as important contextual themes that may have facilitated or impeded action from the intervention. For example, HCP in Lusaka (significant benefit of the intervention) described an existing mechanism for constructive feedback on referrals between facilities, which was aided by the introduction of uniform monitoring equipment. In comparison, HCP from both peripheral and tertiary facilities in Zomba (no benefit of intervention) described negative concerns about referral, such as a lack of system to alert the recipient hospital of the pending transfer resulting in patients being refused admission. HCP in peripheral facilities in Mbale (no benefit of intervention) reported that referrals were reduced following the intervention, since pre-eclampsia could now be managed in the community, which was encouraged by the tertiary facility.

## Discussion

This paper describes the mixed-methods evaluation of implementation alongside a pragmatic, stepped-wedge RCT in ten low- and middle-income sites. We have demonstrated that the CRADLE intervention was delivered appropriately. All clusters demonstrated improved availability of vital signs equipment after the intervention, with increased vital signs measurements in both our quantitative and qualitative analysis. Acceptability of the intervention was good as shown by the high proportion of facilities using the device at 6 and 12 months after implementation and triangulated with the qualitative findings. Referral rates were reduced in the majority of clusters which correlated with qualitative findings. Overall, we have shown no correlation between process measures within domains and no correlation between individual domains and the primary outcome.

Implementation fidelity varied between sites. As this was a pragmatic trial, it was prospectively decided that whilst fidelity would be measured, it would not be used to address and change implementation problems during the trial. This was to ensure generalisability of trial findings in future scale-up, which would likely have limited capacity for detailed monitoring and feedback. The balance between delivering an intervention with high fidelity and adapting to context is widely recognised [18]. We adhered to specific components of training to ensure that delivery was similar across eight countries. However, we demonstrated that it was possible to adapt the delivery model of training whilst maintaining a high proportion of training (as described above for Gokak, India where the research team led all training).

Examples of studies that explain the selection of implementation measures and analyse them alongside primary outcomes are scarce, especially in low-resource settings within the confines of limited infrastructure, research capacity and funds. This paper demonstrates that evaluation of simple implementation process measures alongside a large-scale pragmatic trial is feasible and useful in describing the quality and quantity of implementation in different sites and exploring the potential mechanisms of impact. This methodology provides valuable learning for future research in LMIC by providing information to inform implementation strategies and scale-up.

Research in other fields (e.g. school education) has demonstrated that higher implementation fidelity is associated with better programme outcomes [53]. We have not shown any correlation with the primary outcome. This trial was powered for the primary composite outcome, not the process outcomes. Therefore, it is possible there was insufficient power to detect a significant relationship, as suggested by the wide confidence intervals. It is also possible that this is due to the validity of the process measures themselves or their combination within domains. In the example of reach, the measurement of women that do not attend health services, and were therefore not exposed to the intervention, was not possible. Instead, a surrogate measure of change of equipment availability was selected. It could be argued that clusters with better resources were most likely to demonstrate benefit due to their capacity to respond. Alternatively, those with poor baseline resources may benefit most from the increase in equipment availability. Measuring exactly how an intervention may exert its effect in different settings is challenging within a pragmatic trial of this size. In addition, to minimise the burden of data collection only a few of the many potentially relevant domains could be assessed, and some single items were used to measure some domains. Future research should explore the relationship between implementation strength and trial outcomes and approaches to integration of data.

A further possible reason is the validity of the primary outcome measure in individual sites. As this is a stepped-wedge RCT, the analysis of individual sites’ data is subject to external factors and temporal trends. Whilst these were adjusted for, the variation between and within sites was greater than anticipated and seasonal trends were evident which could not be adjusted for. Due to the scale and setting of the trial, other outcomes such as diagnosis of pre-eclampsia or sepsis were not collected. Despite this, the validity of the CRADLE VSA as an accurate, robust, useful tool is maintained. Mixed-method follow-up of use of the device at 6 months to 1 year after implementation is a strength of this study and supports the sustainability of the intervention. In addition, the proportion of devices that were broken or missing was lower than our sites report for previous existing equipment. Adoption was greater in sites that had higher proportions of HCP trained or more active CRADLE champions or local research teams to support the device. This suggests that these would be important factors for future scale-up.

The strengths of this study are the predefined choice of theoretically-based, predominantly objective, quantitative measures to test hypothesised mechanisms of action. Additional strengths are the integration of qualitative and quantitative measures to triangulate findings and the pragmatic approach to data collection from many routine data sources. Funding restrictions meant the process evaluation and implementation were led by the same research team. This is a possible source of bias, although efforts were made to reduce this by undertaking the initial framework and qualitative analysis prior to analysis of the primary outcome. Whilst the diverse settings of this trial are a strength, the number of sites, resource constraints and the simultaneous delivery alongside a stepped-wedge trial design (with strict intervals for implementation) meant that there was limited capacity to collect additional data in response to early findings.

The success of the intervention is dependent on HCP capacity to change clinical management, particularly in response to an abnormal result. The physical and geopolitical environment within which the intervention is delivered is therefore key. A recent systematic review identified that just 41 RCTs undertaken in sub-Saharan Africa across all health specialities describe any element of context [54]. This study selected a number of quantitative measures of health system infrastructure similar to others in the field [12, 55] and combined this with qualitative review of clinical management and referral pathways. However, these simple measures inadequately described the complexities of these multiple health systems, their clinical pathways and readiness for change [56, 57].

## Conclusions

Evaluation of implementation and integration of results with health outcomes is recommended by the Medical Research Council [18], yet there is insufficient guidance or example of a suitable methodology. To our knowledge, this is the first implementation process evaluation alongside an effectiveness trial that has evaluated implementation using a mixed-methods approach and integrated these with the primary outcome with the aim of understanding differences between multiple low-resource sites. We have demonstrated the successful selection of measures to describe implementation and explore mechanism of action that were feasible. However, the lack of correlation within domains and with the primary outcome suggest that future trials should consider taking further account of the ability of sites to respond, particularly when considering trials of diagnostic tests rather than direct therapeutic interventions. Measurement across all sites was necessary for comparison of implementation. Future research should consider the addition of in-depth analysis in a restricted selection of sites, for example, into clinical care pathways and factors that inform decision-making and deviation from protocols, to explain the effect of complex interventions.

## Additional file

Additional file 1: **Table S1.** Effect of the intervention on referrals in individual clusters. **Table S2.** Effect of the intervention on the primary outcome in individual clusters. Figure S1. Thresholds that trigger the traffic light early warning system on the CRADLE Vital Sign Alert (DOCX 6918 kb)

## Abbreviations

BP: Blood pressure
HCP: Health care professional
LMIC: Low-middle-income countries
RCT: Randomised controlled trial
VSA: Vital Sign Alert
