# Supplementary material for: Exploring the effect of implementation and context on a stepped-wedge randomised controlled trial of a vital sign triage device in routine maternity care in low-resource settings
Source: Implement Sci. 2019 Apr 18;14:38. doi: 10.1186/s13012-019-0885-3 (PMC6471783; doi:10.1186/s13012-019-0885-3)
Supplement: Supplementary file 1 — Table S1. Effect of the intervention on referrals in individual clusters. Table S2. Effect of the intervention on the primary outcome in individual clusters. Figure S1. Thresholds that trigger the traffic light early warning system on the CRADLE Vital Sign Alert (DOCX 6918 kb) [file 13012_2019_885_MOESM1_ESM.docx]

**Supplementary information**

**Figure S1: Thresholds that trigger the traffic light early warning system on the CRADLE Vital Sign Alert**


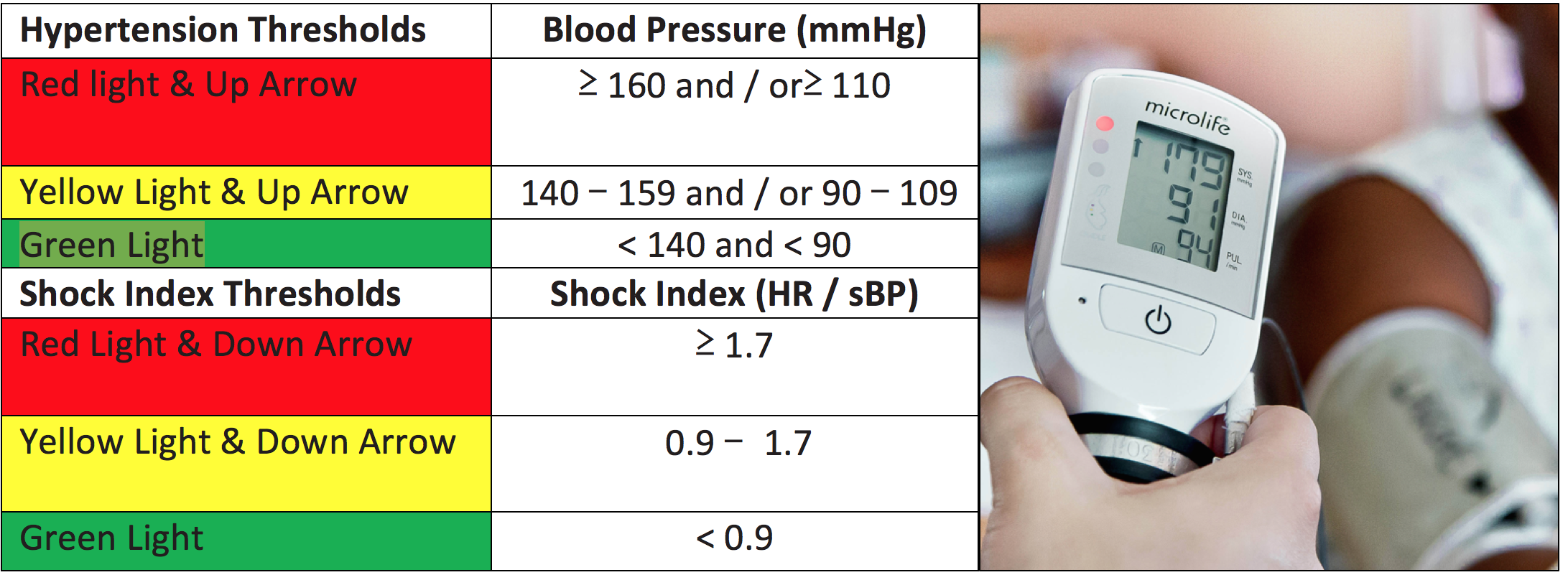


**Table S1 Effect of the intervention on referrals in individual clusters**

| Site | Proportion of women referred to higher level care pre-intervention^ψ^ | Proportion of women referred to higher level care post-intervention^ψ^ | Unadjusted Comparison |
| --- | --- | --- | --- |
|  | N (% of women seen)) | N (% of women seen)) | Odds Ratio (95% CI) |
| Freetown | 45 (1.79%) | 37 (1.11%) | 0.62 (0.40-0.95) |
| Kampala | 302 (*) | 173 (*) | - |
| Ndola | 158 (2.74%) | 123 (2.13%) | 0.56 (0.44-0.71) |
| Lusaka | 1122 (8.3%) | 471 (2.9%) | 0.33 (0.30-0.37) |
| Gokak | 144 (1.12%) | 1306 (15.3%) | 15.95 (13.39 – 18.99) |
| Cap Haitien | 42 (18.19%) | 54 (14.6%) | 0.77 (0.49-1.20) |
| Mbale | 159 (1.46%) | 186 (1.58%) | 1.08 (0.88-1.34) |
| Addis Ababa | 532 (7.59%) | 423 (6.34%) | 0.82 (0.72-0.94) |
| Zomba | 61 (1.64%) | 57 (0.50%) | 0.30 (0.21-0.43) |
| Harare | 521 (8.31%) | 555 (7.48%) | 0.89 (0.79-1.01) |
| All Sites | 2784 (3.7%) | 3212 (4.4%) | 0.89 (0.39-2.05) |

***** Unable to collect denominator data of all women presenting to maternity care.

^ψ^ Recorded for a one-month period immediately prior to implementation, and a one-month period three months after implementation.

**Table S2 Effect of the intervention on the primary outcome in individual clusters**

| Site |  | Pre-intervention | Post-intervention | Adjusted Odds ratio (95% CI)  (p value) |
| --- | --- | --- | --- | --- |
| Addis Ababa | Rate per 10,000 deliveries  (n/N) | 90·2  220/24390 | 89·7  99/11039 | 0·88 (0·70-1·09)  (p=0·23) |
| Cap Haitien | Rate per 10,000 deliveries  (n/N) | 82·1  63/7670 | 108  78/7240 | 0·37 (0·23-0·59)  (p<0·001) |
| Freetown | Rate per 10,000 deliveries  (n/N) | 328  69/2106 | 190  413/21700 | 0·60 (0·50-0·72)  (p<0·001) |
| Gokak | Rate per 10,000 deliveries  (n/N) | 69·4  80/11531 | 37·9  43/11345 | 0·84 (0·65-1·08)  (p=0·18) |
| Harare | Rate per 10,000 deliveries  (n/N) | 95·7  333/34814 | 72·8  26/3569 | 0·98 (0·76-1·26)  (p=0·85) |
| Kampala | Rate per 10,000 deliveries  (n/N) | 73·9  184/24886 | 81·2  836/102931 | 1·32 (1·06-1·65)  (p=0·02) |
| Lusaka | Rate per 10,000 deliveries  (n/N) | 39·4  190/48252 | 14·5  109/75224 | 0·45 (0·33-0·63)  (p<0·001) |
| Mbale | Rate per 10,000 deliveries  (n/N) | 43·8  162/37003 | 40·4  95/23499 | 1·28 (1·04-1·57)  (p=0·02) |
| Ndola | Rate per 10,000 deliveries  (n/N) | 74·3  62/8343 | 81·0  150/18526 | 1·34 (0·98-1·83)  (p=0·07) |
| Zomba | Rate per 10,000 deliveries  (n/N) | 124  600/48243 | 183  255/13922 | 2·09 (1·78-2·47)  (p<0·001) |
| All sites | Rate per 10,000 deliveries  (n/N) | 79·4  1963/247238 | 72·8  2104/288995 | 1·13 (0·85-1·51)  (p=0·40) |

*adjusted for cluster effect, time from start of study; (with an interaction between cluster and time so that each cluster had its own underlying time trend), and total time on the randomized intervention
